# Perinatal depression trajectories and child development at one year: a study in China

**DOI:** 10.1186/s12884-024-06330-4

**Published:** 2024-03-06

**Authors:** Yuan Zhu, Xiaoyu Li, Junyu Chen, Wenjie Gong

**Affiliations:** 1 School of Nursing, Nanjing University of Chinese Medicine, Nanjing, Jiangsu China; 2 HER Team and Department of Maternal and Child Health, Xiangya School of Public Health, Hunan, China; 3 Department of Psychiatry, University of Rochester, Rochester, New York USA; 4 Institute of Applied Health Research, University of Birmingham, Birmingham, UK; 5 Xiangya School of Public Health, Central South University, 172 Tongzipo Road, Yuelu District, Changsha, 410006 Hunan, China

**Keywords:** Trajectories, Perinatal depression, Child development, Perinatal mental health

## Abstract

**Background:**

The objective of the current study was to investigate the correlation between trajectories of maternal perinatal depression (PND) spanning from early pregnancy to one year postpartum and developmental delays observed in one-year-old children.

**Methods:**

The dataset under examination encompassed 880 women who took part in a mother-child birth study conducted in China. Latent class growth analysis (LCGA) was employed to identify patterns in Edinburgh Postnatal Depression Scale (EPDS) scores of women, spanning from early pregnancy to one year postpartum. To assess the neurodevelopment of one-year-old children, a Chinese version of the Bayley Scale of Infant Development (BSID-CR) was employed. Logistic regression was employed to explore the association between PND trajectories and developmental delays in children, with appropriate covariate adjustments.

**Results:**

The trajectories of maternal PND identified in this study included a minimal-stable symptom group (*n* = 155), low-stable symptom group (*n* = 411), mild-stable symptom group (*n* = 251), and moderate-stable symptom group (*n* = 63). Logistic regression analysis revealed that mothers falling into the moderate-stable symptom group exhibited a notably heightened risk of having a child with psychomotor developmental delays at the age of one year.

**Conclusions:**

The findings drawn from a representative sample in China provide compelling empirical evidence that bolsters the association between maternal PND and the probability of psychomotor developmental delays in children. It is imperative to develop tailored intervention strategies and meticulously design mother-infant interactive intervention programs for women with PND.

## Background

Perinatal depression (PND) is defined as a non-psychotic depressive episode of mild to major severity that occurs during pregnancy or up to one year postpartum [1]. The prevalence of PND shows significant variation between high-income countries (HICs) and low- and middle-income countries (LMICs), with a combined prevalence of 11.4% in HICs and 13.1% in LMICs [2]. In China, the estimated pooled prevalence of PND is 16.3% [3], surpassing the average prevalence in LMICs. PND is linked with reduced maternal social support, an increased risk of subsequent maternal depressive episodes, and adverse effects on infant health, including low birth weight and various indicators impacting childhood morbidity and mortality [4, 5]. Additionally, women in LMICs with PND are more likely to experience a more challenging illness trajectory compared to those in HICs [4].

The etiology of PND is complex and multifaceted, involving an interplay between biological, environmental, and symptomatic factors. It is important to note that PND can present differently in each woman, with variations in onset, duration, and symptom configuration. To better understand the chronicity and severity of PND, it is crucial to conduct longitudinal studies using repeated measurement and trajectory analysis. New modeling methods, such as latent class growth analysis (LCGA) and growth mixture modeling (GMM), have been developed to investigate the heterogeneity trajectory of maternal depressive symptoms [6, 7]. The existing studies had described three-six trajectory groups [8, 9], but they have some limitations. Some assess maternal depression symptoms from late pregnancy to less than six months postpartum, while others track symptoms for several years after childbirth. Few studies evaluate the complete trajectory of PND from early pregnancy to one year postpartum. Second, the collection of depression data in these studies has a wide span and a limited number of time points, which may overlook maternal depression symptoms during specific periods. Additionally, most studies are conducted in HICs and pay limited attention to low-income populations. Consequently, the generalizability of the conclusions is uncertain.

The most receptive stage of a child’s development occurs between conception and the age of one [10], during this time the brain exhibits a high level of plasticity and rapid synapse formation, which play an important role in children’s development of motor, emotional, and cognitive abilities throughout their lifespan [11]. Despite the benefits of this in optimal conditions, adverse experiences during this period can have long-lasting and damaging effects on the development of the child [12]. Studies have investigated the impact of maternal depression on child development, but there are limitations. Firstly, the research on the negative effects of depressed mothers on their children has yielded inconsistent results [13–15]. Secondly, studies often rely on cross-sectional data. Moreover, most studies have primarily focused on HICs. Even though depression may act similarly regardless of context, the impact of PND is likely to be magnified in LMICs due to infants being exposed to a greater range of adversities and risk factors, compared to the situation in HICs [12]. Therefore, it is of great significance to explore the relationship between PND trajectories in LMICs and the growth and development of one-year-old children based on longitudinal data.

China’s considerable population of women undergoing pregnancy and childbirth accentuates the significance of investigating perinatal mental health. Exploring the relationship between PND and child development within this context is pivotal. Understanding how maternal mental health influences child development in China can yield insights into potential intergenerational effects and shape strategies for fostering healthy child development. We previously explored the trajectory of maternal depression from early pregnancy to six weeks postpartum, but did not continue tracking beyond one year postpartum, nor did we observe the impact of maternal depression on child development [16]. The current study addresses two gaps in the existing literature. Primarily, we aim to scrutinize the trajectory of PND in China, spanning from early pregnancy to one year postpartum, through meticulous monitoring. Furthermore, we examine whether these PND trajectories independently correlate with child developmental delays at one year, while considering variables like maternal age at birth, education status, mental health history, family history of mood disorders, income level, and marital satisfaction.

## Methods

This study has been conducted following the Declaration of Helsinki, the protocol has been approved by the Ethics Committee of the College of Xiangya Public Health at Central South University, and its registration number is No. ChiCTR-OOC-17,013,766.

### Settings and participants

The data for the current study were collected as part of a study of mother-child births in China [16]. From 2016 to 2018, 1126 pregnant women (less than 13 weeks of gestation) were enrolled in two public maternal and child healthcare hospitals in Hunan Province, China. A woman was considered eligible if she was less than 13 weeks of gestation, 18 years of age or older, and capable of completing questionnaires in Chinese. During the follow-up process, patients exhibiting strong suspicion of moderate to severe depression upon assessment will be referred to the psychiatric department. Patients requiring medication based on the psychiatric assessment will be excluded from the study. Each participant provided informed consent.

### Procedure

The specific collection points are as follows: the recruitment visit occurred before 13 weeks of gestation (T1), subsequent prenatal visits were scheduled at 17–20 weeks gestation (T2), 21–24 weeks gestation (T3), 31–32 weeks gestation (T4), and 35–40 weeks gestation (T5). Postnatal visits were conducted at 1 week postpartum (T6), 6 weeks postpartum (T7), 6 months postpartum (T8), and 12 months postpartum (T9). Participants received a mobile survey link one week before their scheduled obstetric examination appointments through an app called “Wenjuanxing”, a professional online survey platform, and completed the survey on their phones by visiting the linked website. Participants who could not complete the online survey before their clinical visit were interviewed face-to-face by nurses during their obstetric visits. Upon enrollment, every woman was given a specific identification number. Entering this number is required before filling out any questionnaire to authenticate the respondent’s identity. We also collect women’s cell phone numbers and names to match for identification. The research team provides assistance with scheduling examinations, purchasing medication, and free health check-ups as rewards for active participation.

### Maternal PND measure

The assessment for PND among participants involved utilizing the Edinburgh Postnatal Depression Scale (EPDS) across nine distinct time points. Specifically designed to recognize symptoms of postnatal depression experienced by mothers within the preceding seven days, the EPDS comprises 10 items, scoring within a range of 0 to 30 [17]. Murray et al. [18] established the validation of antenatal employment of the EPDS. Our study implemented the validated Chinese iteration of the EPDS [19]. To identify pregnant and postpartum women with elevated symptom levels, we applied a cut-off score of 13. This threshold exhibited a sensitivity of 0.66 (95% confidence interval 0.58 to 0.74) and a specificity of 0.95 (0.92 to 0.96) [20].

### Child measures

We utilized a Chinese version of the Bayley Scale of Infant Development (BSID-CR) to evaluate the neurodevelopment of children at the age of 1 year. The BSID-CR was widely used in China and showed satisfactory performance [21, 22]. Specially trained psychologists blinded to the children’s exposure information conducted the tests. BSID-CR measurement results primarily include the following two indicators: (1) Mental Development Index (MDI), which is a tool for assessing children’s cognitive development, including language, generalization, classification, memory, and social skills; (2) Psychomotor Development Index (PDI), which assesses children’s psychomotor skills, such as muscle coordination and gross and fine manipulation abilities. Individuals whose MDI or PDI scores fall below 79 are categorized as experiencing abnormal development, while scores between 80 and 119 indicate normal development, and scores of 120 or higher suggest positive developmental progress [21].

### Covariates

Among the covariates included in the multivariate analysis are maternal, family, and child characteristics. Maternal characteristics were age at birth, educational level (junior high school or below, high school, bachelor’s, postgraduate), and history of mental health problems (no vs. yes). Family characteristics included a history of mood disorders (like depression and bipolar disorder; no vs. yes), income level (0-2000, 2001–5000, 5001–10,000, > 10,000), and marital satisfaction (very satisfied, satisfied, and dissatisfied). Child characteristics were continuous variables, which included the height, weight, and head circumference of a one-year-old child.

### Statistical analysis

To calculate maternal PND trajectories, we used LCGA implemented with PROC TRAJ in SAS 9.4 (SAS Institute, Cary, North Carolina). This group-based semiparametric method allows us to identify distinct clusters of individual trajectories within the population [23]. The missing data were handled using PROC TRAJ under the missing-at-random assumption, whereby individuals with missing data were assigned to the trajectory group that was deemed to be the most likely based on available data [24]. Based on Mughal’s research [25], we hypothesized the identification of four distinct trajectory groups, and we tested models fitting two to five classes consecutively. We compared fit indices for each model to determine the best fit for the data. These fit indices included the Bayesian Information Criterion (BIC), with a value closer to zero indicating a better model fit, and the bootstrapped parametric likelihood ratio test (BPLRT), which compares the model with K classes to a model with K-1 classes and provides a *p*-value representing whether there is a statistically significant improvement in fit for the model with one more class [26]. High entropy was used as an index of classification accuracy, with a probability value above 0.8 indicating an accurate classification [27]. The proportion of participants within each class was also considered, with no fewer than 5% of participants expected to be in a single class [28]. To examine the associations between PND trajectories and child developmental delays, logistic regression models were conducted with dummy-coded latent trajectory class groups as the predictors and each Bayley-BR score grade as the outcome, in separate analyses per Bayley-BR outcome, adjusting for covariates.

## Results

### Characteristics of participants

Between September 2016 and March 2018, out of the 1,126 pregnant women included, a total of 880 participants completed at least three depression assessments from pregnancy through one year postpartum. Among these, 47 participants completed the questionnaire three times, 48 individuals completed it four times, 64 took part in five completions, 82 engaged in six completions, 192 completed it seven times, 209 completed it eight times, and 238 completed it nine times. Among the 246 lost to follow-up, 189 individuals refused to continue participating after completing the first assessment, 41 did not complete the three assessments due to miscarriage or relocation, and 16 declined to continue after completing two assessments. Women who completed three or more EPDS assessments tended to have higher education levels, a pattern that extended to their husbands (*P*<0.05). Additionally, this group of women was more likely to be pregnant for the first time. (*P*<0.05). Among the 880 participants, the average age at the time of enrollment was 28.22 years old (SD = 4.40), with over two-thirds holding a bachelor’s degree or higher (68.4%). In terms of personal monthly income, approximately half of the participants earned between 2001 and 5000 yuan, making up 50.7%, while 24.4% reported being unemployed. A significant majority expressed satisfaction with their spousal relationships (82.7%). Only a small percentage of participants and their families had a history of depression (2.5% and 2.3%, respectively). The mean levels of EPDS at different time points were: M = 8.58, SD = 3.99 (time 1); M = 8.18, SD = 4.26 (time 2); M = 7.98, SD = 4.01 (time 3); M = 7.87, SD = 4.04 (time 4); M = 7.79, SD = 3.92 (time 5); M = 7.02, SD = 4.04 (time 6); M = 7.38, SD = 4.11 (time 7); M = 7.70, SD = 3.08 (time 8); M = 6.61, SD = 2.84 (time 9). The total sum of scores was also employed as a continuous variable for statistical analysis, showing good internal reliability with Cronbach’s alphas ranging from 0.77 to 0.85.

Of the 880 participants included in the trajectory analysis, 431 completed BSID-CR and were included in the logistic regression analysis. The average levels of the length, weight, and head circumference of the one-year-old babies were 70.89 cm (SD = 0.57), 8.37 kg (SD = 0.18), and 43.53 cm (SD = 0.23), respectively. Four-fifths of the children’s one-year-old BSID-CR cognitive development index was medium, 9.28% was excellent, and 4.41% was poor. In terms of the one-year-old motor development index, the medium level accounted for 79.58%, the poor accounted for 15.78%, and the 4.64% was excellent. Independent samples t-test and chi-square test were used to compare the differences in baseline characteristics between participants with and without missing BSID-CR data. The results showed that participants without BSID-CR data had significantly lower education levels and monthly income compared to participants with BSID-CR data (*p* < 0.01). There was no statistically significant difference between the two groups of participants in terms of whether they had medical insurance, personal or family history of depression, and marital relationships.

### Trajectories of PND symptoms

Latent class growth analyses specifying two-five group models were estimated (Table 1). The four-group model was accepted as the final model after inspecting all indexes and tests of model fit. The diagonal posterior probabilities for the three-group model were slightly higher than the four-group model, but it had a lower BIC value. And the five-class model subdivided the sample into smaller groups (one additional group of 4.93%) that did not improve the classification of subjects.

**Table 1:** Model fit information for LCGA specifying two-five groups

#Class	BIC	BPLRT *P* value	Entropy	Proportions within each Group, n (%)				
				Group 1	Group 2	Group 3	Group 4	Group 5
2	-17052.27	<0.001	0.95	546(62.04%)	334(37.96%)			
3	-16731.70	<0.001	0.91	272(30.94%)	451(51.26%)	157(17.80%)		
**4**	**-16583.35**	**<0.001**	**0.89**	**155(17.59%)**	**411(46.68%)**	**251 (28.52%)**	**63(7.21%)**	
5	-16553.58	<0.001	0.84	83(9.43%)	286(32.54%)	298(33.91%)	169(19.19%)	43(4.93%)

**Fig. 1:**
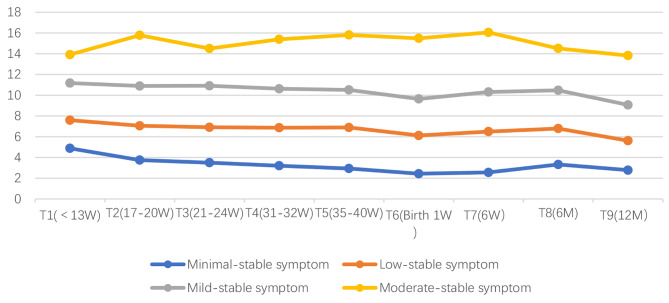
Four-group trajectory of EPDS scores across the period (*n* = 880). W: week; M: month

Figure 1 illustrates the average trajectory of each group from pregnancy to one year postpartum. The groups are as follows: the minimal-stable symptom group (*n* = 155; 17.59%), which includes women who reported minimal symptoms of depression; the low-stable symptom group (*n* = 411; 46.68%), the largest trajectory, comprises women who reported consistently low levels of depression symptoms; the mild-stable symptom group (*n* = 251; 28.52%), which includes women with a higher level of symptoms that approach but do not exceed the severity cut-off of 13; and the moderate-stable symptom group (*n* = 63; 7.21%), the smallest trajectory, which comprises of women who are likely to be diagnosed with clinical depression.

### PND trajectories and child development

Next, we examined predictors of trajectory group assignment, with the minimal-stable symptom group serving as the reference group. The results of these regression models are presented in Table 2. The moderate-stable symptom group was associated with lower PDI grades [β(95% CI) = -1.61(-2.80, -0.41), *p*<0.01], but there was no association with MDI grades at one year. The other groups were not significantly associated with any BSID-CR developmental delays. The negative effects of the moderate-stable symptom group on PDI at one year persisted even after adjusting for the aforementioned factors known or suspected to be associated with child development.

**Table 2:** Regression models for PND trajectories predicting BSID-CR scores at one year

PND trajectories	Psychomotor Development Index (PDI)		Mental Development Index (MDI)	
	β (95% CI)	*P* value	β (95% CI)	*P* value
Unadjusted model				
Minimal-stable symptom	0.00 (ref)	0.00 (ref)	0.00 (ref)	0.00 (ref)
Low-stable symptom	-0.42(-1.016, 0.22)	0.20	-0.07(-0.85, 0.72)	0.87
Mild-stable symptom	-0.15(-0.82, 0.53)	0.67	0.39(-0.46, 1.24)	0.37
Moderate-stable symptom	-1.80(-2.95, -0.64)	**<0.01**	-0.94(-2.09, 0.21)	0.11
Adjusted model				
Minimal-stable symptom	0.00 (ref)	0.00 (ref)	0.00 (ref)	0.00 (ref)
Low-stable symptom	-0.35(-1.01, 0.32)	0.31	-0.08(-0.80, 0.79)	0.98
Mild-stable symptom	-0.06(-0.68, 0.66)	0.87	0.42(-0.47, 1.31)	0.36
Moderate-stable symptom	-1.61(-2.80, -0.41)	**<0.01**	-1.02(-2.21, 0.18)	0.09

## Discussion

### Main findings

To the best of our knowledge, this study is the first to investigate the maternal depression trajectories from early pregnancy to one year postpartum, focusing on the complete PND cycle and utilizing dense data from nine-time points in China. We identified four-group PND trajectories: minimal-stable, low-stable, mild-stable, and moderate-stable symptom groups. We find an association between moderate-stable PND symptoms and children’s motor development at 1 year, but there was no association with mental development.

### Possible mechanisms of the findings

We have identified four distinct PND trajectories in our study. These findings align with previous research conducted by Other scholars [29, 30], where similar trajectories were observed and characterized as exhibiting temporal stability with a linear trend. With the exception of the moderate-stable symptom group, the trajectories of the remaining three groups all fell below the EPDS cut-off value of 13. This suggests that only a small proportion of women within these groups are experiencing clinical depression. However, it’s worth noting that the minimal-stable symptom group had the smallest representation among the three groups, accounting for only 17.59%. In contrast, the low-stable symptom group comprised a larger population at 46.68%. This trend aligns with the findings reported by Luoma et al. [31], who observed a distribution of 18% and 53% respectively in similar groups. These findings indicate that only a relatively small proportion genuinely escape from experiencing emotional challenges. Indeed, feelings of sadness can be encountered by numerous perinatal women due to a complex interplay of factors, encompassing hormonal fluctuations, physical transformations, disturbances in sleep patterns, and individual psychological attributes [32]. The trajectory exhibiting moderately stable symptoms accounted for a minority, around 7.21% of the sample, aligning closely with the 8.3% found in Kuo et al.‘s study [29]. This indicates a distinct subset of women experiencing persistent clinical depression throughout pregnancy and the initial postpartum phase. This might be associated with economic and social factors, obstetrical history, and biological factors, lifestyle and history of mental illness, as well as the interaction between these factors [33]. In our prior work, our team utilized GMM to analyze data across seven distinct time points [16] and identified two trajectories labeled as “antenatal high” and “postnatal high,” accounting for only 5.1% and 4.9% of the trajectory distribution. However, these trajectories were not observed in the current study. In this study, we utilized LCGA, obtaining trajectory class probabilities exceeding 0.7, resulting in a more balanced distribution, higher model fit, and more robust research findings. Additionally, two additional follow-up time points were included, significantly increasing the dataset used for trajectory analysis compared to previous research. Thus, it is evident that tracking time points, sample size, and research methodology may influence the variation in trajectories of PND symptoms. Moving forward, exploring optimal tracking schemes and comparative research methodologies will enable us to precisely elucidate the trajectories of PND.

To the best of our knowledge, this study is the first to investigate the relationship between PND trajectories and child developmental delays in one-year-old offspring in China. We find an association between moderate-stable PND symptoms and children’s motor development. Maternal hormonal secretion and immune system dysfunction occurring during prenatal depression can potentially impact the development and functionality of children’s motor regions through two distinct pathways: Fetal-Maternal Hypothalamic Pituitary Adrenal Axis Dysregulation and Uterine Artery Resistance [34]. These pathways may significantly influence the child’s motor development and coordination abilities [35]. Study [36] found that infants born to mothers with depression had lower gray matter volume in the frontal lobe, cingulate gyrus, and insula, and these infants had fewer white matter fiber tract connections in the cingulum and superior longitudinal fasciculus, which may affect their motor planning, execution. Mothers experiencing depression may face challenges in providing stimulation, which could hinder infants’ motor learning and development [37, 38], such as encouraging crawling and exploring the environment, or limited interaction time and quality. We did not find any association between moderate-stable PND symptoms and children’s mental development. However, some research indicates that the chronicity of depression contributes to poorer cognitive or language outcomes in toddlers and young children [39–41]. The inconsistency in our findings could be attributed to several factors. Developmental abnormalities in infants might not become immediately evident at specific time points. Abnormalities in motor development are particularly noticeable and sensitive during infancy, while the impact on intellectual development tends to become more significant as children grow [42]. Moreover, diverse parenting styles and levels of engagement could influence the development of children’s cognitive and language skills [43]. Even in the presence of depression, a nurturing and enriching parenting environment has the potential to mitigate potential negative effects [35, 44]. In conclusion, the relationship between maternal depression and children’s development is intricate and multifaceted, influenced by a complex interplay of biological, psychological, social, and environmental factors. The inconsistencies in research findings underscore the critical importance of thoroughly considering these factors when exploring the effects of maternal depression on children’s mental development. Moreover, further studies that specifically focus on various developmental stages are essential to better understand the impacts of PND on children’s overall well-being.

### Limitation

This study has several limitations. First, the response rate for the survey was relatively low, with around 22% of participants not completing all nine screenings. Only half of the women who participated in the trajectory survey collected data on their children, which may lead to biased results. Secondly, the assessment of PND relied on self-reports, lacking information on clinical diagnosis, a common limitation in psychological research. Despite the EPDS being a widely used screening tool for PND, it cannot substitute clinical diagnostic outcomes. Moreover, this study involved EPDS measurements at nine time points, potentially leading to regression dilution bias. Depression values recorded at later stages are more prone to being lower than the actual values, possibly resulting in an underestimation of depression severity. Thirdly, the relevant variables investigated in the study were relatively limited, especially the lack of inclusion of duration of exclusive breastfeeding and the variables related to social support, which is highly correlated with the development of other children. Whatmore, it’s important to note that this is an association observed in the study, and while it suggests a connection between maternal depressive symptoms and child development, it doesn’t necessarily imply a direct causal relationship.

### Implications and recommendations

Initiating screening and management of PND immediately upon confirming pregnancy is of utmost importance. A comprehensive team comprising doctors, mental health specialists, obstetricians, and pediatricians should devise tailored plans for individuals at high risk of depression and implement effective strategies to diminish both the occurrence and progression of depressive symptoms. These strategies may encompass psychological therapy, medication, or other appropriate interventions aimed at alleviating depressive symptoms. Additionally, advocating for mother-infant interactive intervention programs can prove invaluable in fostering positive parent-child relationships. Family members can significantly contribute by providing emotional support and practical assistance, thereby aiding women in managing their depression and facilitating the healthy growth and development of infants and young children.

## Conclusions

In this study, we identified four-group PND trajectories: minimal-stable symptom group, low-stable symptom group, mild-stable symptom group, and moderate-stable symptom group. Logistic regression analysis indicated that mothers in the moderate-stable symptom group were at a significantly higher risk of having a child with psychomotor developmental delays at one year. It is imperative to develop tailored intervention strategies and meticulously design mother-infant interactive intervention programs for women with PND.

## Data Availability

The datasets generated during and/or analyzed during the current study are available from the corresponding author upon reasonable request.

## Abbreviations

PND: Perinatal depression
LCGA: Latent class growth analysis
EPDS: Edinburgh Postnatal Depression Scale
BSID-CR: Chinese version of the Bayley Scale of Infant Development
HICs: High-income countries
LMICs: Low- and middle-income countries
GMM: Growth mixture modeling
MDI: Mental Development Index
PDI: Psychomotor Development Index
BIC: Bayesian Information Criterion
BPLRT: Bootstrapped parametric likelihood ratio test
